# CNN Based Detectors on Planetary Environments: A Performance Evaluation

**DOI:** 10.3389/fnbot.2020.590371

**Published:** 2020-10-30

**Authors:** Federico Furlán, Elsa Rubio, Humberto Sossa, Víctor Ponce

**Affiliations:** Instituto Politécnico Nacional, Centro de Investigación en Computación, Ciudad de México, México

**Keywords:** convolutional neural network (CNN), rock detection, machine learning, planetary exploration, remote sensing

## Abstract

An essential characteristic that an exploration robot must possess is to be autonomous. This is necessary because it will usually do its task in remote or hard-to-reach places. One of the primary elements of a navigation system is the information that can be acquired by the sensors of the environment in which it will operate. For this reason, an algorithm based on convolutional neural networks is proposed for the detection of rocks in environments similar to Mars. The methodology proposed here is based on the use of a Single-Shot-Detector (SSD) network architecture, which has been modified to evaluate the performance. The main contribution of this study is to provide an alternative methodology to detect rocks in planetary images because most of the previous works only focus on classification problems and used handmade feature vectors.

## 1. Introduction

Research interest in planetary missions centered on exploring on-site regions of Mars or the Moon is increasing. Remarkable examples of this are the next NASA mission to Mars with a new Rover generation (NASA, 2020) or the recent Chinese (Amos, 2020) and Arabian launches. Projects that have reached singular success are the exploration missions performed by geologist robots. Their main task is to retrieve samples that could give clues about the past of the terrain conditions of vital importance for future missions. A serious problem in these missions originates from data transmission latency, which is the time needed to send information from the robot location back to Earth, in contrast to a reduced time window for this assignment. Therefore, the robot must be able to detect objects of interest like rocks autonomously. A typical method used for object detection is through image processing. But conditions typically encountered in planetary environments, like arid terrains devoid of any kind of vegetation, as well as similar color and texture scenarios, results in poor performance of conventional image processing methods that usually are not adequate to different lighting conditions. This makes it necessary to experiment with models capable of handling information with uncertainty and effective in recognizing objects of interest with tolerance to the disturbances present in the captured images, such as Artificial Neural Networks (ANN).

In Gao et al. (2014) several approaches to detect objects in planetary terrains are introduced, suggesting that neural networks could provide promissory results. In many research works found in literature, ANN and recently, Convolutional Neural Networks (CNN), have demonstrated astonishing results in a diversity of problems related to object recognition, surpassing the performance of other approaches. Typical works deal meanly with images that focus on scenes taken from houses, offices, or cities. Other works are specialized in medical or biological images. However, the number of articles that employ CNN to process planetary terrain images or lands with characteristics alike is reduced.

Results from testing two CNN architectures, along with a Visual Geometry Group Neural Network (VGG) type and a Residual Neural Network (Resnet) for rock classification are reported in Li et al. (2020), where an approach called transfer learning is employed, which consists of using the trained weights of a model processed over a large amount of data as the initial weights of the CNN. The second training model named fine-tuning adjusts the CNN weights with a smaller dataset of the object of interest. They reported extraordinary results with an accuracy of 100%, by using a VGG16. Also, they compare the results with conventional methodologies, like Histogram Oriented Gradients (HOG) or Scale-Invariant Feature Transform (SIFT), plus a Support Vector Machine (SVM) that reaches a humble accuracy of around 63% and 75%. They used, as the dataset, images captured from the Curiosity mission, Nevertheless, images are trim and show only a rock.

In Furlán et al. (2019), a methodology to detect rocks using a CNN is presented, where a U-net, which is a convolutional neural network introduced in Ronneberger et al. (2015), was adapted to segment panoramic images taken in a Mars-like environment located on Earth. An F1-score of 78% while improving the inference latency of the algorithm is reported. The results were satisfactory and similar to other methodologies.

This work is aimed to evaluate the performance of some CNN's models for rock detection tasks, in a Mars-like environment, demonstrating that a CNN can be an alternative to conventional image detection techniques, due to their inherent advantage for handling the uncertainty found typically in unexplored terrains, paving the way for ambitious exploration traversals. Indeed, a combination of CNNs with neuromorphic computing, based on memristor technologies are gaining attention as future intelligent computing platforms for image detection due to their ultra-low power consumption and implementation on integrated circuits (Amravati et al., 2018) and (Chen et al., 2019). A combination of CMOS-camera with a neuromorphic chip, running CNN based algorithms for image recognition is expected to become the next step for planetary Rover missions.

## 2. Materials and Methods

Recent advances in object detection that use CNN models have achieved successful results with different datasets, like COCO (Lin et al., 2014) or Pascal VOC (Everingham et al., 2010). COCO and Pascal VOC are datasets consisting of images taken in different scenarios, focused on detecting objects like cars, people, cats, dogs, among other daily life objects. Due to those promising results, we considered testing the performance of such CNN architectures with unstructured objects typical in outdoor environments.

We are interested in experimenting with the CNN architectures in a Mars-like environment where the main task is to detect rocks. The methodology proposed uses a Single-shot Multibox Detector (SSD) to detect rocks, which are objects of interest in an exploration mission.

### 2.1. Single-shot Multibox Detector

**Figure 1:**
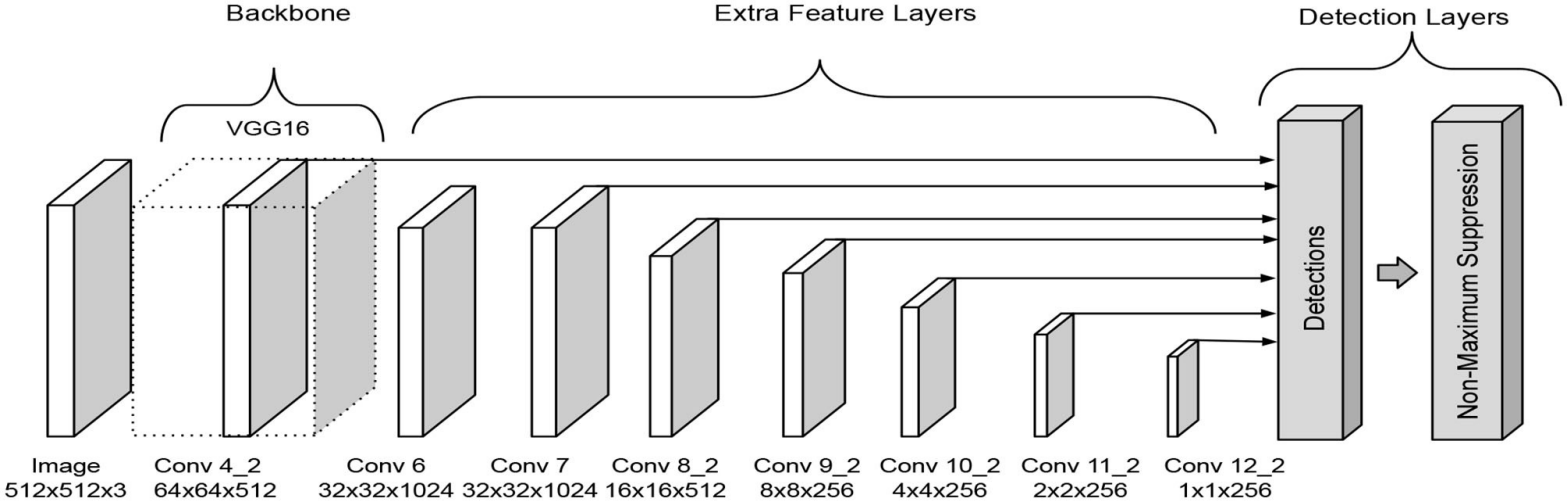
SSD architecture as presented in Liu et al. (2016).

The Single-shot Multibox Detector was introduced in 2016 (Liu et al., 2016). The architecture is formed by three parts, a backbone followed by a series of convolutional feature extraction layers and the detection layers. It is required to apply a non-maxima suppression process to obtain the correct output, which are the corresponding predicted boxes in the image, see Figure 1.

In the original paper, the backbone corresponds to a truncated VGG-16 network that works as a feature extraction phase. The extra feature extraction layers decrease gradually in size to make predictions on different scales. The detection layers align the top feature layers with bounding boxes that have multiple predefined scales and ratios. The predictions obtain for each bounding box related to the feature layer are the offset of the position of the bounding box and a confidence value that means whether a class is present in the region or not. An advantage of this architecture is that it makes predictions at multiple scales, which improves the detection in comparison with other models like Faste R-CNN.

The function of the extra feature extraction layers is to generate default bounding boxes using convolution filters. For each feature layer, a small kernel operates to obtain a membership value for each class or an offset measured relative to the default bounding box position, depending on the convolutional layer.

A set of default bounding boxes is associated with each feature layer where the predictions will be made. So, each bounding box will produce *c* score values, where *c* is the number of classes to be detected, and 4 location offsets relative to the initial box position. For example, an *m* × *n* feature map produces a total of (*c* + 4)*k* filters that are applied around each location, where *k* is the number of boxes, generating (*c* + 4)*kmn* outputs.

The loss function is a weighted sum of two functions.

(1) $$\begin{array}{l} L\left(x,c,l,g\right)=\frac{1}{N}\left(L_{conf}\left(x,c\right)+\alpha L_{loc}\left(x,l,g\right)\right) \end{array}$$

The localization loss function (*L*_*loc*_(*x, l, g*)) estimates the closeness between the predicted box (*l*) and the ground truth box (*g*). It measures the difference in the center location (*cx, cy*) and the width (*w*) and height (*h*) of the predicted box relative to the ground truth box. It uses a Smooth L1 norm and α is a weight term.

The confidence loss function (*L*_*conf*_(*x, c*)) compares the predicted classes with the ground truth classes for each bounding box. It uses a softmax function. For a detailed explanation of the loss functions consult (Liu et al., 2016).

### 2.2. Dataset

The images used with the CNN model are from (Furgale et al., 2012) created by the Autonomous Space Robotics Lab (ASRL) from the University of Toronto. The original dataset is a compilation of more than 50,000 images captured during a 10-kilometer traverse in the Mars analog site on Devon Island located in Canada. The dataset is not labeled. Hence to avoid the laborious task of manually mark every image, we separated an image each five frames ending with a dataset of 5172 images.

During the labeling process, we discarded images that didn't display rocks. In the end, the final dataset has 1,600 labeled images that include a total of 8,372 objects labeled as rocks. Then, we divided the dataset into 1,280 images for training and 320 for validation. To examine the performance of the CNN model, we selected a different dataset for testing.

**Figure 2:**
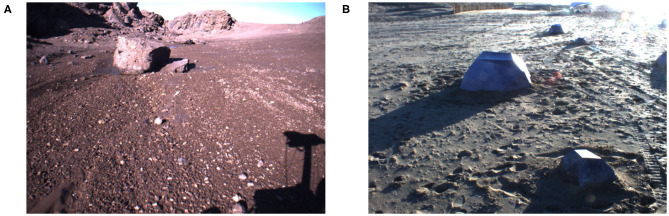
Sample images form dataset **(A)** Devon Island (Furgale et al., 2012) and **(B)** Katwijk beach (Hewitt et al., 2018).

We used The Katwijk beach planetary rover dataset (Hewitt et al., 2018) that uses artificial models of rocks of different sizes and distribute them around a beach to resemble a planetary terrain. We manually labeled 331 images to estimate the generalization ability of the models. In Figure 2, we show an image from each dataset.

### 2.3. Proposed CNN

We introduce two modified versions of the original SSD architecture presented in Liu et al. (2016). We resized the dataset images to 512 × 512, which is the input size of the models. ReLu is the activation function used in all convolution operations.

The scales are parameters required in the detection layers, which are obtained using the next equation:

(2) $$\begin{array}{l} s_{k}=s_{min}+\frac{s_{max}-s_{min}}{m-1}\left(i-1\right) \end{array}$$

where *s*_*min*_ = 0.1 and *s*_*max*_ = 1.06, *m* is the number of predictions layers for all models. In this work, *m* = 7 is considered, the first scale is set as 0.04, and *i* is the number of scales needed in the model. The scales used in all models are [0.04, 0.1, 0.26, 0.42, 0.58, 0.74, 0.9, 1.06].

The models proposed makes predictions over 7 layers, the aspect ratios used for all models are the same as in the original paper (Liu et al., 2016). The aspect ratios for prediction layers 1, 6 and 7 are $\left[1,2,\frac{1}{2}\right]$ and for prediction layers 2, 3, 4 and 5 are $\left[1,2,\frac{1}{2},3,\frac{1}{3}\right]$.

**Figure 3:**
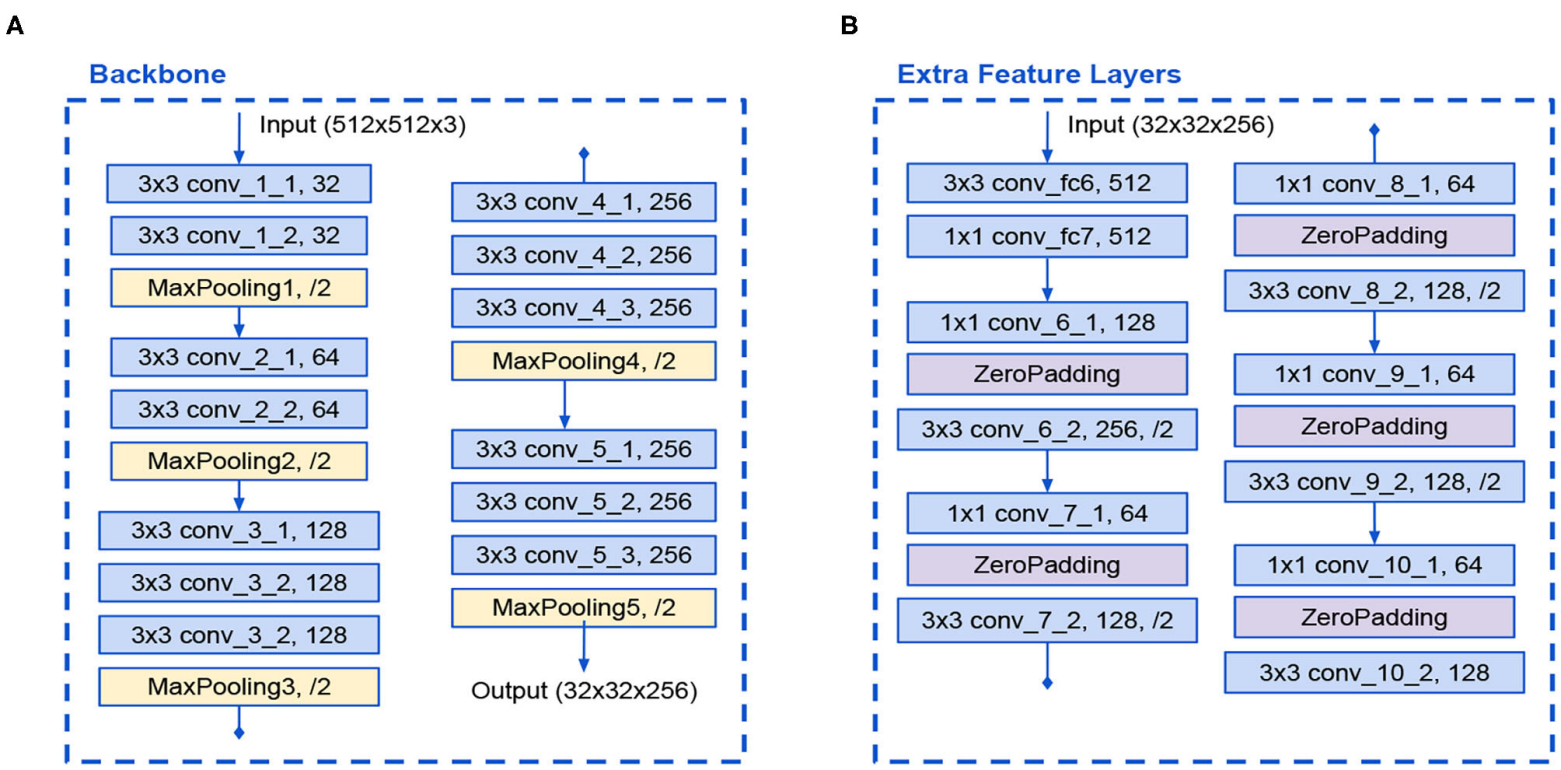
**(A)** Modified VGG16 backbone and **(B)** Modified Extra Feature Layers in SSD A architecture.

The first introduced model is a modified version of the original SSD architecture that reduces the number of filters in the VGG16 backbone in half. This backbone has 13 convolutional layers with 3 × 3 kernels. The input size is reduced from 512 to 32 due to 5 max-pooling operations. A detailed diagram of the Backbone is presented in Figure 3. Additionally, the Extra Feature Layers also reduced its filters in half and is formed by 12 convolutional layers with 1 × 1 and 3 × 3 kernels with strides of 2 that caused feature maps dimension reduction. A detailed diagram of the Extra Feature Layers is presented in Figure 3.

**Figure 4:**
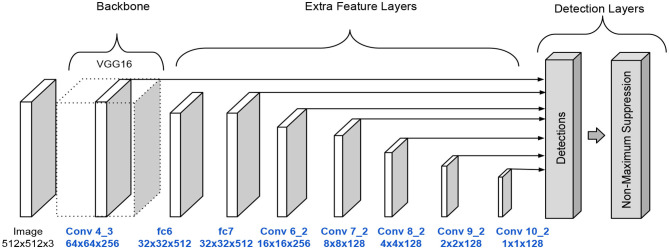
SSD architecture version A.

These modifications lessen the number of parameters. The full SSD A architecture is shown in Figure 4.

**Figure 5:**
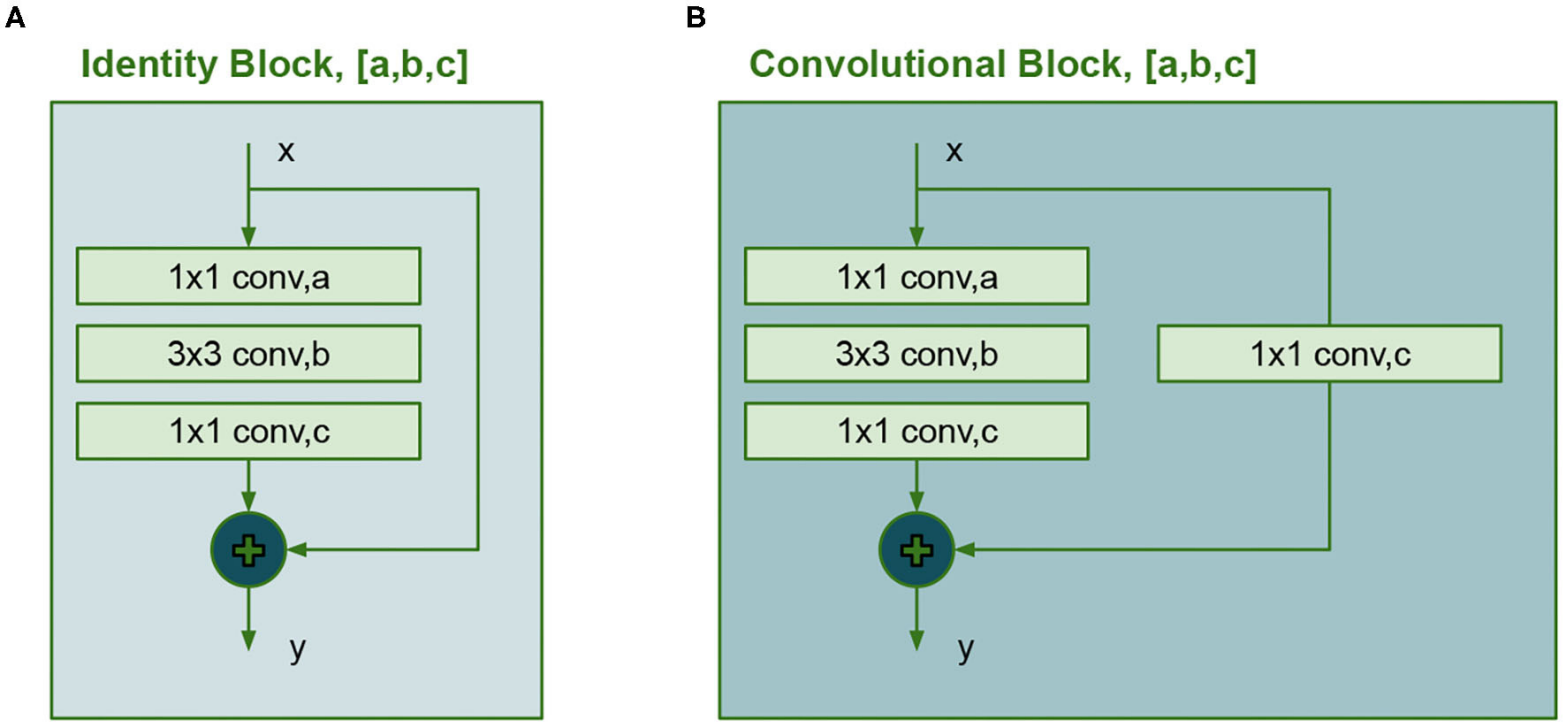
Building blocks for the ResNet50, **(A)** Identity block and **(B)** Convolutional block.

Previous works like (He et al., 2017) used ResNet configurations as a backbone to improve the performance for detections and instance segmentation tasks but require big datasets for training since its large number of trainable parameters. In the second model, the VGG16 net is replaced with a convolutional network inspire in ResNet50. The new backbone uses two types of building blocks known as identity block and convolutional block. Their unique property is the shortcut connection, which consists of an add operation between an early convolution and the final convolution. A detailed diagram of these blocks is shown in Figure 5. The identity block has 3 convolutional layers and the convolutional block has 4 layers.

**Figure 6:**
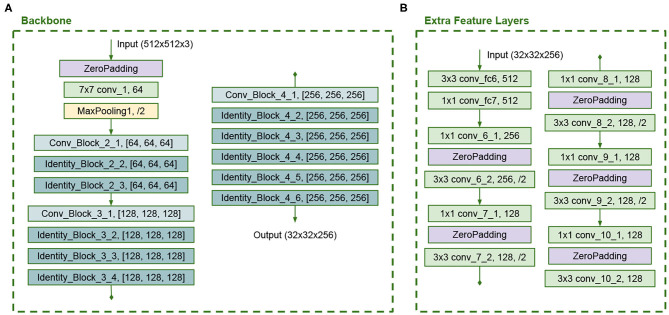
**(A)** Modified ResNet50 backbone and **(B)** Modified Extra Feature Layers in SSD B architecture.

**Figure 7:**
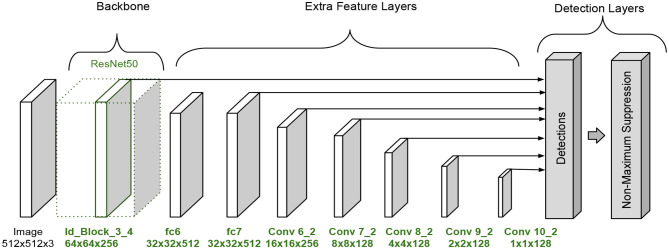
SSD architecture version B.

The backbone architecture is similar to the ResNet50, but we use the same number of filters in all convolutions in each building block and truncate it at stage 4. In the original ResNet50 model, the last convolution has more filters than the other convolutions in each block. Figure 6 presents the backbone configuration. This backbone has 43 layers. The Extra Feature Layers are configured as in Figure 6 and have 12 layers. These modifications in the model reduce the number of trainable parameters. The full SSD B architecture is shown in Figure 7.

An original SSD configuration serves as a baseline for comparing performance with the introduced models. The original model is named SSD O in tables and graphics and shares the same configuration as the SSD A model. The only difference is the number of filters in the convolutional operations.

The code from (Ferrari, 2018), which is a Keras implementation of the original SSD architecture, was modified to run with Tensorflow 2.2. The generator function was transformed to read CSV files from the label datasets. The corresponding architectures presented in this article were developed as functions for the training process. Each training process took about 18 h of time execution, using an Intel i9 computer equipped with 64 Gb of RAM and two GPU cards installed, to complete the job with a learning rate of 0.001.

**Figure 8:**
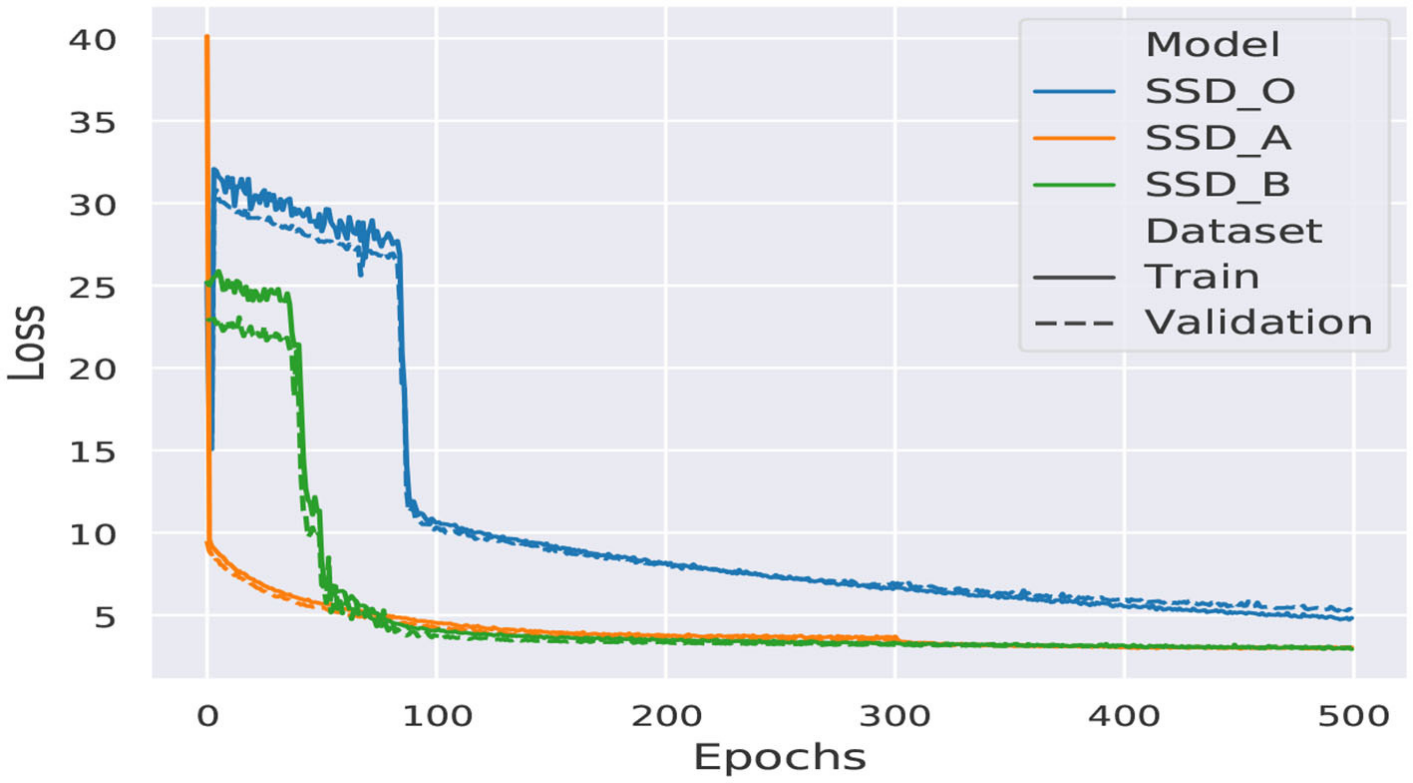
SSD architectures training curves.

We utilized stochastic gradient descent (SGD) during 500 epochs to adjust the parameters during the training process. We used data augmentation to change the images with one of four transformations, which could be photometric distortion, expansion, random crop, or random horizontal flip. The intention of using data augmentation is to evade overfitting while training the models. The training process of a model requires only one execution to generate a weights file, that later will be loaded in the model to implement the inference task. Each execution will produce similar results, but not the same since the weights are randomly initialized using a He normal distribution. The resulting learning curves are shown in Figure 8.

## 3. Results

Table 1 shows a comparison of the number of parameters within the architectures. The number of parameters is associated with the complexity of the net and the inference time. The inference time denotes how long does the CNN take to produce a prediction. A remarkable characteristic of the SSD architecture is that it delivers what can be considered real-time performance. Table 1 shows the average inference times for each model for each model running over the training computer.

**Table 1:** Comparison of the number of parameters and inference time.

**Model**	**Number of parameters**	**Inference time (milliseconds)**	**FPS**
SSD Original - VGG16	24,088,664	55.36	18
SSD A - VGG16	6,320,632	38.70	25
SSD B - ResNet50	10,088,664	39.01	25

The mean average precision per dataset (Train, Validation, and Testing) is listed in Table 2. This value represents how many target objects are predicted or detected by a CNN. The higher the number obtained, the network performance is better. This value is bounded to the [0,1] interval.

**Table 2:** Comparison of the mean average precision.

	**mAP**			
**Model**	**Train**	**Validation**	**Testing**	**Standard deviation**
SSD Original -VGG16	0.815	0.604	0.233	29.46%
SSD A -VGG16	0.627	0.520	0.174	23.68%
SSD B - ResNet50	0.451	0.353	0.253	9.90%

**Figure 9:**
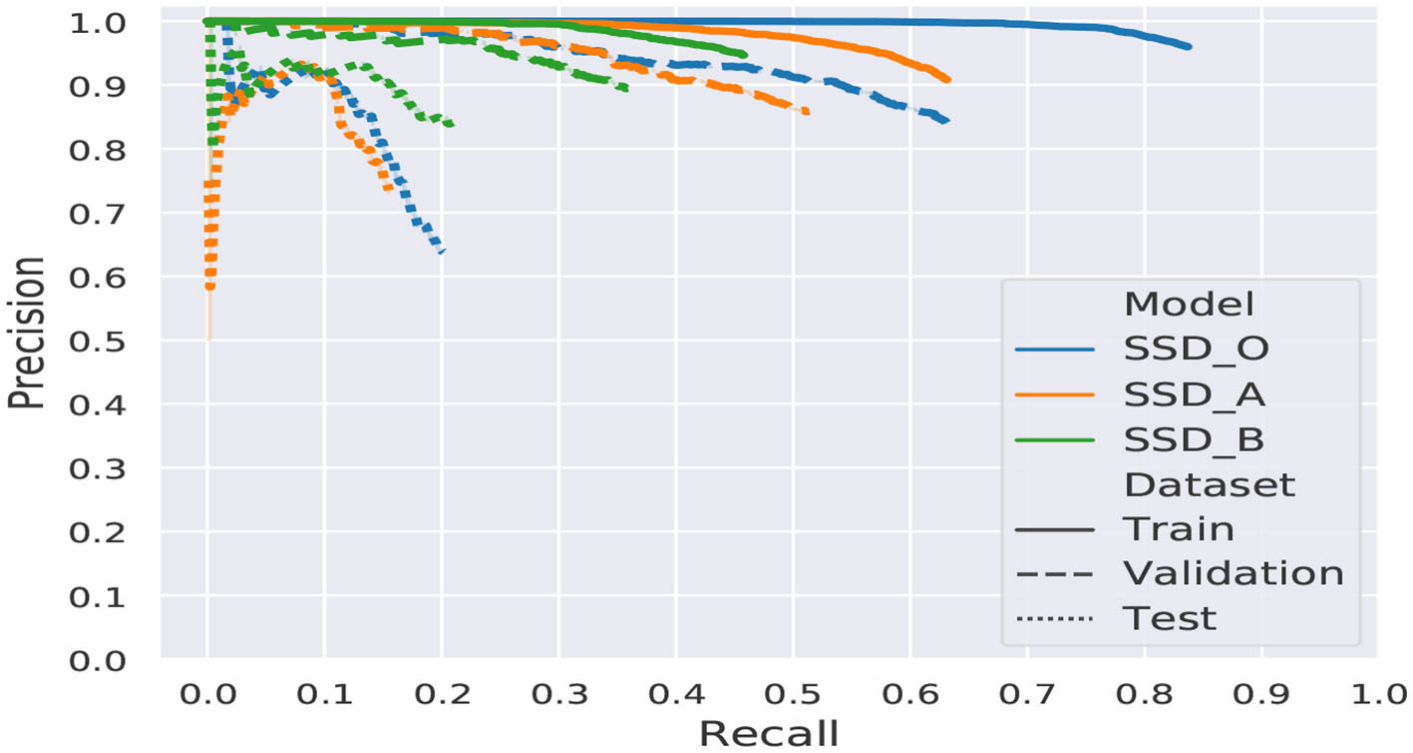
Comparative graphic of the mAP.

Additionally, the graphs of the mAP for each model and dataset are shown in Figure 9.

The original architecture shows signs of overfitting, caused by a large number of trainable parameters, more than twice the number of parameters of the proposed models. Another factor that contributes to the overfitting is the reduced amount of images of the dataset. Since this model posses a large number of parameters, it shows an undesired behavior conducting to memorize the training data, which results in a high mAP for training and validation but a significant drop for the testing dataset.

The results showed that there is plenty of room for improvement. Model A achieved better results for training and validation, while model B scored better in testing. Hence to determine which model is better, we need to remember that most of the planetary applications are focus on exploring unknown environments to find valuable scientific information.

Therefore we need a model capable of generalizing, which means, be capable of achieving high-grade performance with unknown data slightly different from the training data. Model B has a lower standard deviation among its mAP over all datasets.

**Figure 10:**
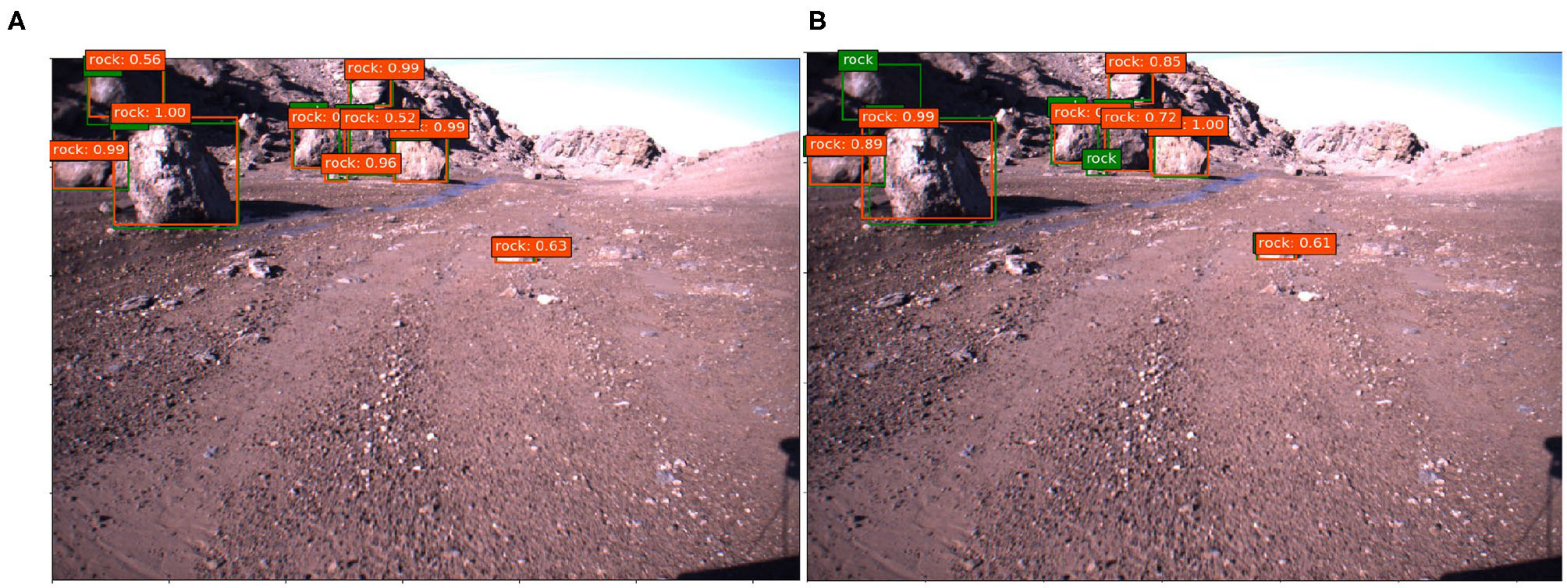
Examples of predictions with Devon Island dataset, **(A)** SSD A and **(B)** SSD B.

**Figure 11:**
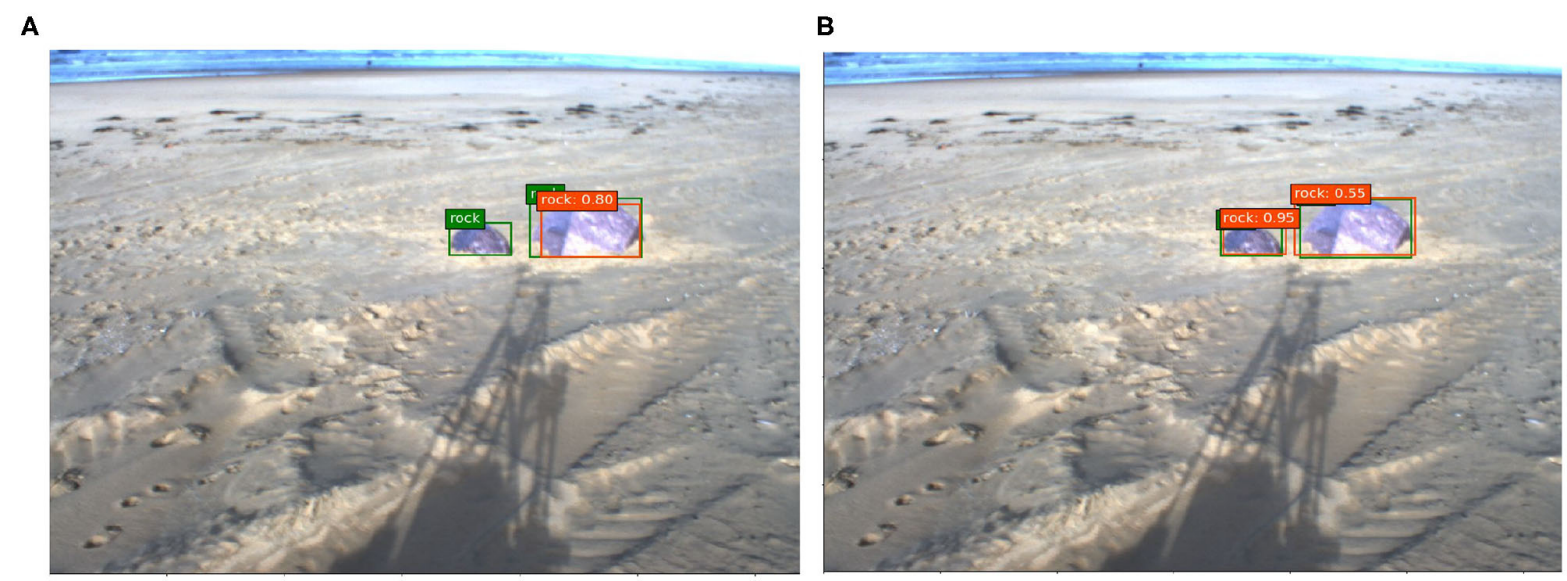
Examples of predictions with Katwijk beach dataset, **(A)** SSD A and **(B)** SSD B.

We show some testing images with their corresponding predictions and ground truths in Figures 10, 11. The predictions made by the network are depicted with a red square along with its confidence value, which means the grade of accuracy that the boxed object is a rock. Lastly, the ground truth is labeled with a green square.

## 4. Discussion

Previous methodologies employed to detect rocks in planetary environments require algorithms that need handmade feature vectors, which are complicated to design and are dependant on expert knowledge and the feature extractors applied that sometimes are not robust. This study evaluates an alternative solution adopting a supervised learning algorithm to avoid selecting feature extractors. Since CNN's are tolerant of translation transformations, and also trained appropriately permit small rotations or scale transformations, it adds a factor of robustness. It could become part of an autonomous navigation system because rocks are the main obstacles for rovers traversals, and with the same algorithm fulfill two functions detecting valuable samples and obstacles.

This methodology can improve while operating in an unknown environment by collecting new images and adding them to the training dataset. The training process can be performed remotely in a high-performance computer and then transmit the weights file to be updated on the operation site. The expected result would be an enhanced performance caused by the new knowledge acquired from the unexplored area. Space exploration missions use remote sensing equipment to broadcast information to a control center. Hence this methodology would be suitable for object detection process.

## Data Availability Statement

The raw data supporting the conclusions of this article will be made available by the authors, without undue reservation.

## Author Contributions

All authors contributed to the conception and design of the study. FF selected the datasets. HS and ER proposed modifications to the CNN architectures. VP wrote the first draft of the manuscript. FF developed the code required for the experiments. All authors contributed to manuscript revision, read, and approved the submitted version.

## Conflict of Interest

The authors declare that the research was conducted in the absence of any commercial or financial relationships that could be construed as a potential conflict of interest. The reviewer JAMC declared a shared affiliation, though no other collaboration, with the authors to the handling Editor.
